# Comparison of enzymatic and bisulfite-based methods for sequencing-based cell-free DNA methylation profiling

**DOI:** 10.3389/freae.2025.1693925

**Published:** 2026-01-16

**Authors:** Mark Ezegbogu, Peter Averill Stockwell, Glen Reid, Ben Brockway, Rajiv Kumar, Euan Rodger, Aniruddha Chatterjee

**Affiliations:** 1 Department of Pathology and Molecular Medicine, Dunedin School of Medicine, University of Otago, Dunedin, New Zealand; 2 Department of Medicine, Dunedin School of Medicine, University of Otago, Dunedin, New Zealand

**Keywords:** cell-free DNA, methylation, cancer, EM-seq, cfRRBS, cfMethyl-Seq

## Abstract

Epigenetic analysis, especially DNA methylation profiling of plasma cell-free DNA (cfDNA), has recently emerged as a promising clinical tool. Choosing the right analytical method is crucial for working with limited cfDNA, ensuring cost-effectiveness, and supporting clinical translation. While bisulfite-based methods have long been the standard for methylation analysis, enzymatic conversion is a potential alternative. However, their comparative performance for cfDNA remains unclear. In this study, we compared enzymatic (EM-Seq) and bisulfite-based (cfRRBS and cfMethyl-seq) methods. EM-Seq showed higher mapping efficiency, broader genomic coverage, and captured more CpGs at low coverage thresholds, while the bisulfite methods had higher conversion rates, lower costs, and better coverage of functional regions like promoters and exons. The bisulfite-based methods also demonstrated superior reproducibility. Overall, cfRRBS offered the best balance of cost, accuracy, and reproducibility. Our findings fill a key gap in cancer epigenetics, outline the strengths and limitations of each method, and provide a practical guide for selecting cfDNA methylation profiling methods in liquid biopsy applications.

## Introduction

DNA methylation is a fundamental epigenetic mechanism in all nucleated cells, with aberrant methylation patterns serving as hallmarks of various diseases, including cancer. Recent advances have established cell-free DNA (cfDNA) methylation analysis as a powerful approach for non-invasive disease detection and monitoring. However, a rigorous evaluation of cfDNA methylation profiling methods is critical for clinical translation. Current approaches for DNA methylation analysis (both sequencing and array-based) predominantly rely on tissue biopsies as the primary DNA source (Sun et al., 2015). Although tissue biopsies are still considered the diagnostic gold standard, they have notable technical limitations. In oncology, for example, their utility is confined to lesions that are both clearly identifiable and safely reachable for surgical collection. Furthermore, tissue biopsies impose significant inconvenience on patients without guaranteeing positive outcomes, thereby raising critical clinical and ethical concerns regarding their risk-benefit assessment (Mannelli, 2019; Overman et al., 2013).

cfDNA addresses these challenges, as its acquisition is minimally invasive, retains the genetic information of nuclear DNA, and captures more local tissue heterogeneity, while demonstrating comparable analytical performance to tissue biopsies (Vrba et al., 2020; Chen et al., 2020; D et al., 2021; Ezegbogu et al., 2024). Although cfDNA analysis encompasses mutation assays, fragmentomics and methylomics, the latter holds excellent potential for future clinical use as methylation changes are pervasive throughout the genome and their potential to serve as early biomarkers for disease pathogenesis (Allen Chan et al., 2013; Baylin et al., 2001; Esteller et al., 2024). The genome-wide distribution of DNA methylation marks in cfDNA enables comprehensive molecular profiling, while their tissue-specific patterns enable origin prediction in liquid biopsy applications.

DNA methylation is measured by the enzymatic or bisulfite conversion of unmethylated cytosines to thymines, and has been discussed extensively in recent reviews (Ezegbogu et al., 2024; Singer, 2019; Wang et al., 2022). In humans, methylation predominantly occurs at CpG dinucleotides, which are often concentrated in CpG-rich regions, such as near gene promoters (Jones, 2012). Reduced representation approaches, such as RRBS (Reduced Representation Bisulfite Sequencing), focus on these CpG-rich regions, which, despite covering approximately 10% of all CpG sites in the human genome (Beck et al., 2021), capture a significant proportion of dynamic methylation changes, particularly in regulatory regions (Deaton and Bird, 2011; Meissner et al., 2005).

In the classic RRBS method, CpGs are enriched for by size-selecting DNA fragments between 40 and 220 bp (Gu et al., 2011); however, this size band coincides with the average size of cfDNA (150 – 200 bp (Fan et al.,2010)), thus making it challenging for traditional RRBS to enrich for CpG-rich regions in cfDNA. Cell-free reduced representation bisulfite sequencing (cfRRBS (Koker et al., 2019)) and cell-free methyl sequencing (cfMethyl-Seq (Stackpole et al., 2022)) overcome these challenges by dephosphorylating the 5′- and blocking the 3′-ends of cfDNA with chain-terminating ddNTPs (in cfMethyl-Seq); these modifications permit the enrichment for CpG-rich regions in cfDNA. These methods are well-documented and optimised for profiling clinical samples (De Wilde et al., 2024; Van Paemel et al., 2021; Li et al., 2023).

In contrast, the enzymatic approach for DNA methylation profiling uses enzymatic conversion rather than a bisulfite-based chemical reaction. Enzymatic Methyl Sequencing (EM-Seq) is the leading enzymatic method that has generated substantial interest recently (Gan et al., 2024; Taylor et al., 2025). In EM-Seq, methylated cytosines are protected using Tet methylcytosine dioxygenase 2 (TET2) and T4-phage beta-glucosyltransferase (T4-BGT). The unmethylated cytosines are ultimately converted to thymines after deamination by APOBEC3A (Taylor et al., 2025). A recently optimised one-tube protocol (referred to as single-enzyme methylation sequencing or SEM-seq (Vaisvila et al., 2024)) leverages a single enzyme for differential cytosine deamination, avoiding the DNA loss risks posed by NaOH denaturation and multi-step bead purifications in traditional EM-Seq workflows. The key advantages of enzymatic methods for cfDNA methylation analysis include mitigating PCR bias, owing to reduced PCR cycle requirements, and preventing DNA degradation associated with bisulfite chemical conversion (Wang et al., 2022; Shen et al., 2019; Lambert et al., 2019).

The integration of chemistry into molecular biology research has enabled the development of different methods for cfDNA methylation profiling. To justify their use, these new approaches are typically benchmarked against gold-standard approaches such as whole genome bisulfite sequencing (WGBS) or reduced representation bisulfite sequencing (RRBS). Unsurprisingly, many of these newer methods outperform standard RRBS, which is not optimised for cfDNA, and surpass WGBS in cost-effectiveness (Stackpole et al., 2022). However, most prior evaluations, including recent comprehensive assessments (Nuttall et al., 2025; Simons et al., 2025), were conducted using sheared genomic DNA as a proxy for plasma-derived cfDNA. While such simulations provide ample input material, they overlook the distinct fragmentation patterns, low abundance, and biochemical constraints inherent to cfDNA. Our study benchmarks enzymatic and bisulfite-based methods, directly on plasma-derived cfDNA under clinically relevant, low-input conditions, thereby filling a key translational gap between methodological performance and real-world clinical utility.

## Methods

### Sample collection

16 mL of blood was collected from two healthy volunteers into Streck cell-free DNA blood collection tubes and processed within 24 h of collection to ensure the integrity of the samples. Whole blood samples were centrifuged at 1,600 x g for 10 min at room temperature to separate plasma from blood cells. The supernatant was then subjected to a second centrifugation at 16,000 x g for 10 min at room temperature to remove residual cellular debris. Each volunteer provided informed consent. This study was approved by an Independent Ethics Committee - Central Health and Disability Ethics Committee (HDEC), Ministry of Health, New Zealand (Ethics approval number: 2022 EXP 12566).

### cfDNA extraction, quality control, and library preparation

cfDNA was extracted from 5 mL of plasma using the QIAamp MinElute ccfDNA Midi Kit according to the manufacturer’s instructions. The extracted cfDNA sample from each volunteer was divided equally into two tubes to generate technical replicates. One replicate pair was used to prepare EM-Seq libraries, and the other for cfRRBS libraries. cfDNA concentration was measured using the Qubit 4 fluorometer, and quality was assessed using an Agilent 2100 Bioanalyser instrument to confirm the absence of genomic DNA contamination. For consistent comparison, 10 ng of cfDNA was used to prepare EM-Seq and cfRRBS libraries.

For the EM-Seq libraries (Vaisvila et al., 2021), only the control DNA was sheared (i.e., CpG methylated pUC19 and unmethylated lambda DNA) to 300 bp using the Covaris M220 focused-ultrasonicator with 130 μL microtube AFA Fibre pre-slit snap-cap tubes. Furthermore, a 2x bead purification protocol was used to purify the adaptor-ligated DNA, then 1.8x, 2x, and 1x bead purification protocols for the TET2-converted, deaminated, and amplified DNA, respectively. 0.1N NaOH was used for deamination. Each library was amplified using the following conditions: 98 °C for 30 s, 10 cycles of 98 °C for 10 s, 62 °C for 30 s, 65 °C for 60 s, and a final extension at 65 °C for 5 min. For the cfRRBS libraries (Koker et al., 2019), the adaptor-ligated DNA was bisulfite-converted twice using the QIAGEN EpiTect Plus bisulfite conversion kit (Stackpole et al., 2022). PCR amplification was performed using the NEBNext Multiplex Oligos for Illumina under the following conditions: 95 °C for 5 min, followed by 16 cycles of 98 ^°^C for 20 s, 65 ^°^C for 15 s, and 72 ^°^C for 45 s, with a final extension at 72 ^°^C for 5 min. Library cleanup was performed using a 2.5x Ampure bead purification protocol (See Figure 1). The reagents used for library preparation are listed in Supplementary Material S1.

**FIGURE 1:**
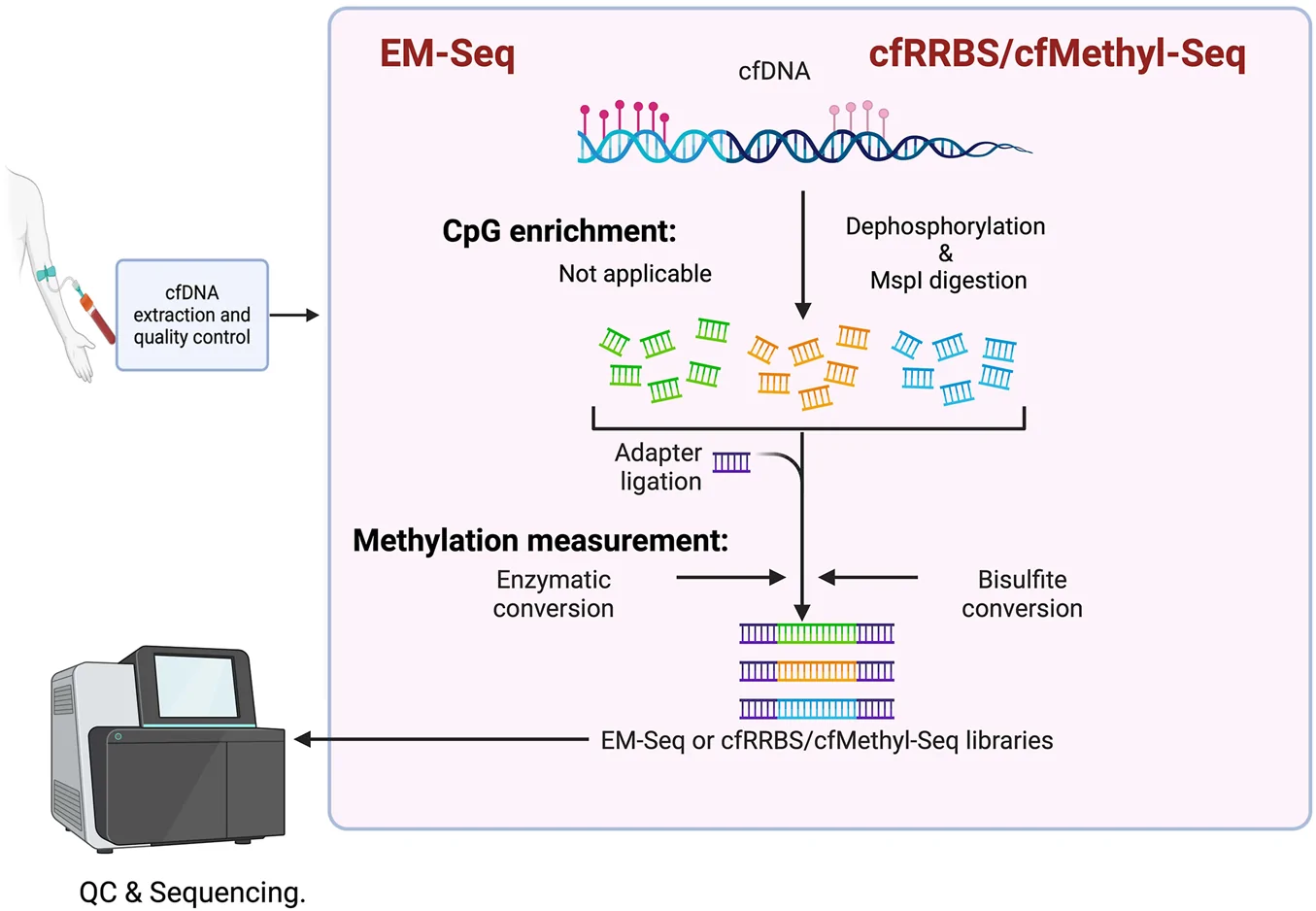
Experimental protocol highlighting the key differences between EM-Seq and cfRRBS/cf-Methylseq. The critical distinctions between EM-Seq and cfRRBS/cfMethyl-Seq lie in their approaches to CPG enrichment and cytosine conversion. In cfRRBS and cfMethyl-Seq, CpG enrichment is achieved by first dephosphorylating the 5′ end of the DNA molecule using recombinant shrimp alkaline phosphatase, followed by MspI digestion. This enables differentiation between DNA fragments flanked by MspI cut sites and those with only one or no MspI cut sites, with the latter degraded by exonucleases. By contrast, EM-Seq interrogates the entire genome and does not require CpG enrichment. The second significant difference is the mode of cytosine conversion. In EM-Seq, unmethylated cytosines are enzymatically deaminated to thymines by APOBEC3A, whereas in cfRRBS and cfMethyl-Seq, cytosines are first bisulfite-converted to uracils, which are subsequently read as thymines following PCR amplification.

### Sequencing and data preprocessing

The EM-Seq and cfRRBS libraries were sequenced on the Illumina NextSeq 2000 platform with 100 bp paired-end and 100 bp single-end modes, respectively. Sequencing services were provided by the Otago Genomics Facility at the University of Otago, New Zealand. A minimum sequencing depth of 10x was targeted for all methods. Bioinformatic processing was performed identically across samples using the following bioinformatics pipeline: quality control was performed using the FASTQC tool (v0.12.1) (Babraham Bioinformatics, 2025), and adaptor/quality trimming was carried out using TrimGalore (v0.6.10) (TrimGalore/Docs, 2025). For the cfRRBS libraries, the–rrbs option was used to remove 2 bp from the 3′ end of the reads. This ensures that the filled-in cytosines introduced during end-repair at MspI cut sites are excluded from downstream methylation calling (Stackpole et al., 2022).

The sequencing reads were aligned to the unmethylated lambda phage DNA and reference human genome (hg19) using Bismark (v0.22.3) (Krueger and Andrews, 2011) with no mismatch allowed (-N 0). Methylation calls were generated with the Bismark methylation extractor (v0.22.3) using the--comprehensive option to combine strand-specific information into context-dependent output files (CpG, CHG, and CHH). The--bedgraph option was applied to produce bedgraph files indicating the genomic positions of detected cytosines and their corresponding methylation states. Unique CpG dyads were quantified using the coverage2cytosine module (v0.22.3) (GitHub, 2025). To ensure fair comparisons across libraries sequenced at different depths, CpG dyad counts were normalised to CpGs per million sequencing reads, representing the number of CpGs retained for an equivalent unit of sequencing effort.

Cytosine conversion efficiency for the EM-Seq and cfRRBS libraries was assessed using the proportion of sequencing reads aligned to the lambda phage DNA, as shown in Equation 1. The conversion efficiency for the EM-Seq libraries was also verified using the CpG-methylated pUC19 DNA spike-in control, as shown in Equation 2. Since the cfMethyl-Seq protocol does not include spike-in controls, the cytosine conversion efficiency for this method was determined by proxy from the proportion of methylated cytosines aligned to the human genome in the CHG, CHH, and unknown contexts. This is expressed mathematically in Equation 3.

$$\text{Cytosine conversion efficiency }\left(\text{Lambda}\right)=100-\left[\frac{CpG+CHG+CHH}{3}\;x\;100\right]$$ (1)

$$\text{Cytosine conversion efficiency }\left(\text{pUC}19\right)=\frac{CpG+CHG+CHH}{3}\;x\;100$$ (2)

$$\text{Cytosine conversion efficiency }\left(\text{proxy}\right)=100-\left[\frac{CHG+CHH+unknown}{3}\;x\;100\right]$$ (3)

### Methylation data analysis

The coverage2cytosine report, containing chromosomal coordinates and methylation information, was used as input for methylation data analysis. Technical reproducibility was assessed using Pearson Correlation and Bland-Altman analysis. Genomic annotation was done using the annotatr R package (v1.32.0) (Bioconductor, 2025). Promoter regions were defined as −5 kb upstream and +1 kb downstream of the Transcription Start Site (Plaisier et al., 2016). CpG site context was assigned using the default annotatr classes: CpG shores were defined as the regions 2 kb flanking CpG islands; CpG shelves were defined as the subsequent 2 kb regions adjacent to the shores; and all remaining genomic segments outside these regions were classified as open sea, or inter-CGI regions (Bioconductor, 2025).

To compare methylation patterns across regulatory regions, we generated meta-profiles centred on transcription start sites (TSS) and CpG islands. TSS coordinates were obtained from the UCSC TxDb.Hsapiens.UCSC.hg19.KnownGene database, and promoter regions were defined consistently defined as −5 kb to +1 kb relative to the annotated TSS. CpG islands were downloaded from the UCSC Table Browser CpG island track (Karolchik et al., 2004). For each sequencing method, CpG sites passing a minimum coverage threshold of 10x were merged across technical replicates, and methylation beta-values were converted to comparable percentage scales (0–100%). For meta-profiling, ±1 kb window were extracted around each TSS and CpG islands; overlapping CpGs were assigned a relative position to the window centre, binned into 50-bp intervals, and mean methylation was calculated per bin per method.

To assess methylation within transcriptionally active promoters, we analysed a panel of ten commonly used housekeeping genes (GAPDH, ACTB, RPLP0, HPRT1, B2M, TBP, PGK1, HMBS, RPL13A, PPIA), selected based on consistently stable expression across tissues (Krzystek-Korpacka et al., 2014; She et al., 2009; de Jonge et al., 2007). Statistical comparisons employed Friedman tests followed by Dunn’s post-hoc tests with Benjamini–Hochberg correction for multiple comparisons. Data processing and visualisation were performed in R (version 4.4.1) using ggplot2 and dplyr.

### Sequencing data for the cfMethyl-Seq method

To evaluate the performance of the cfRRBS and EM-Seq with an additional bisulfite-based method, we have used cfMethyl-Seq data from two healthy plasma samples obtained from the European Genome-Phenome Archive (study ID: EGAS00001006020). The raw fastq files (normal_plasma_1N and normal_plasma_10N) were processed as described by Stackpole et al. (2022). Briefly, these processing steps include UMI and fixed sequence removal, adaptor and quality trimming, alignment, and methylation calling. More details are provided in the ‘sequencing and data preprocessing’ section. Downstream analyses (correlation and CpG site annotation) were performed in the same manner as for EM-Seq and cfRRBS for consistency.

## Results

### Comparison of methylation sequencing, mapping efficiency, cytosine conversion and cost

All libraries produced high-quality sequencing data, with Phred Score >30, corresponding to an error rate of 0.001%. As a whole-genome-based method, EM-Seq yielded an average of 296 million paired-end reads (i.e., 134 million and 162 million unique read pairs per replicate) (Table 1; Supplementary Table S1). For cfMethyl-Seq, 160 million paired-end reads (i.e., 80 million and 84 million unique read pairs per replicate) were analysed, while for cfRRBS, we have generated ∼48 million single-ended reads (23 million and 31 million reads per replicate). The mapping efficiency is the proportion of sequencing reads that map uniquely to the reference genome. EM-Seq showed the highest mapping efficiency with mean unique alignments exceeding 80% (80.5% and 81.4% per replicate). In contrast, the bisulfite-based methods showed lower mapping efficiencies: 59.5% for cfMethyl-Seq (60.9% and 57.9% per replicate) and 53.6% for cfRRBS (53.8% and 53.1%) (Table 1).

**TABLE 1:** Technical comparison of EM-Seq, cfRRBS, and cfMethyl-Seq.

Parameter	EM-seq	cfRRBS	^a^cfMethyl-seq
Sequencing reads	147.6M read pairs	47.4M reads	80M read pairs
Uniquely aligned reads	120.6M read pairs	25.4M reads	47.6M read pairs
Mapping efficiency	81.7%	53.6%	59.5%
^b^Cytosine Conversion Efficiency	98.4%	99.7%	99.8%
^c^On-target rate	Not applicable	95.6%	97.7%
^d^Cost per library preparation	US$96	US$97	NA
^d^Cost for sequencing	US$1322	US$345	NA
Total cost per patient methylome	US$1418	US$442	NA

The data in Table 1 are the average values for each library preparation method across the sample replicate pairs (See Supplementary Table S1).

^a^ External dataset.

^b^ The conversion efficiency was determined by measuring the mean percentage of methylated cytosines in the CpG, CHG, and CHH, contexts of the lambda phage DNA, spike-in control. The conversion efficiency of cfMethyl-Seq was determined using the non-CpG, methylation information from the alignment to the human genome. The conversion efficiency of the EM-Seq libraries was further verified using the CpG-methylated pUC19 control. We obtained 95.2% and 95.4% CpG methylation per replicate (Expected pUC19 CpG methylation: 96 – 98% (Vaisvila et al., 2021; Vaisvila et al., 2024; New England Biolabs, 2025)).

^c^ The on-target rate is the proportion of sequencing reads that fall within the RRBS genome (i.e., reads that start and end at MspI-cut (CCGG) sites).

^d^ The cost per library preparation comprises phlebotomy consumables, quality control (DNA quantification and bioanalyser assessment), and library preparation. The total cost per patient methylome represents the costs of library preparation and sequencing. A detailed cost breakdown is available in Supplementary Material S2.

NA: not available.

In DNA methylation analysis, the efficiency of cytosine conversion (either by enzymatic or chemical reactions) is crucial for accurately distinguishing true CpG methylation. We assessed conversion efficiency using the percentage of methylated cytosines in the CpG, CHG, and CHH contexts of the lambda phage DNA spike-in control. The cfMethyl-Seq libraries did not include a spike-in control; direct assessment of conversion efficiency was not possible for this method. Instead, we estimated conversion efficiency for cfMethyl-Seq by measuring non-CpG methylation levels (CHH and CHG contexts) in the Bismark alignment report for the human genome as an alternative approach. All three methods demonstrated excellent conversion efficiency (>98%). Notably, the bisulfite-based methods (cfRRBS and cfMethyl-Seq) achieved superior conversion rates exceeding 99.5%, as shown in Table 1.

For clinical implementation and to enable methylome analyses for larger patient cohorts within a finite budget, it is essential to consider the balance between cost and data yield. Based on our calculations, EM-Seq incurs a total cost of US$1,519 per sample, comprising US$118 for library preparation and US$1,401 for sequencing at the depth used in this study. In contrast, cfRRBS costs US$450 per patient methylome, with US$86 for library preparation and US$366 for sequencing. For cfMethyl-Seq, the sequencing cost is comparable to cfRRBS, but as the libraries were prepared externally, we could not determine the exact library preparation cost. Nevertheless, our results indicate that cfRRBS and cfMethyl-Seq offer substantially more cost-effective cfDNA methylome profiling options than whole-genome scale methods such as EM-Seq, even at a conservative sequencing depth of 14.5x coverage.

### Comparison of usable data between EM-Seq, cfRRBS and cfMethylseq

In methylation sequencing experiments, coverage filtering is commonly performed after processing the sequencing reads to ensure the robustness of methylation calling, leaving usable data for further analysis and interpretation. We have defined usable data in two ways: 1) the proportion of uniquely aligned sequencing reads for downstream analysis (measured by mapping efficiency), and 2) the number of CpG dyads detected by each method at different coverage thresholds.

We evaluated the coverage efficiency of each method by examining CpG dyad retention across minimum coverage thresholds (1x to 50x). In the raw CpG counts, EM-Seq yielded the largest number of CpGs at shallow depth (mean 27 million at 1x), reflecting its genome-wide sampling strategy (Supplementary Table S1a). However, EM-Seq exhibited the steepest attrition in CpG retention as coverage thresholds increased, with a 4-fold reduction between 1x and 10x. In contrast, cfRRBS and cfMethyl-Seq began with fewer total CpGs (3.8 million and 6.8 million at 1x, respectively) but showed substantially more gradual loss across thresholds (Supplementary Figure S1). Because sequencing depth differed across libraries, we normalised counts to CpGs per million reads, which confirmed the same pattern (Figure 2; Supplementary Table S1b). EM-Seq maintained the greatest CpG density at 1x and 5x, but cfRRBS and cfMethyl-Seq retained markedly more CpGs per million reads at ≥10x coverage thresholds.

**FIGURE 2:**
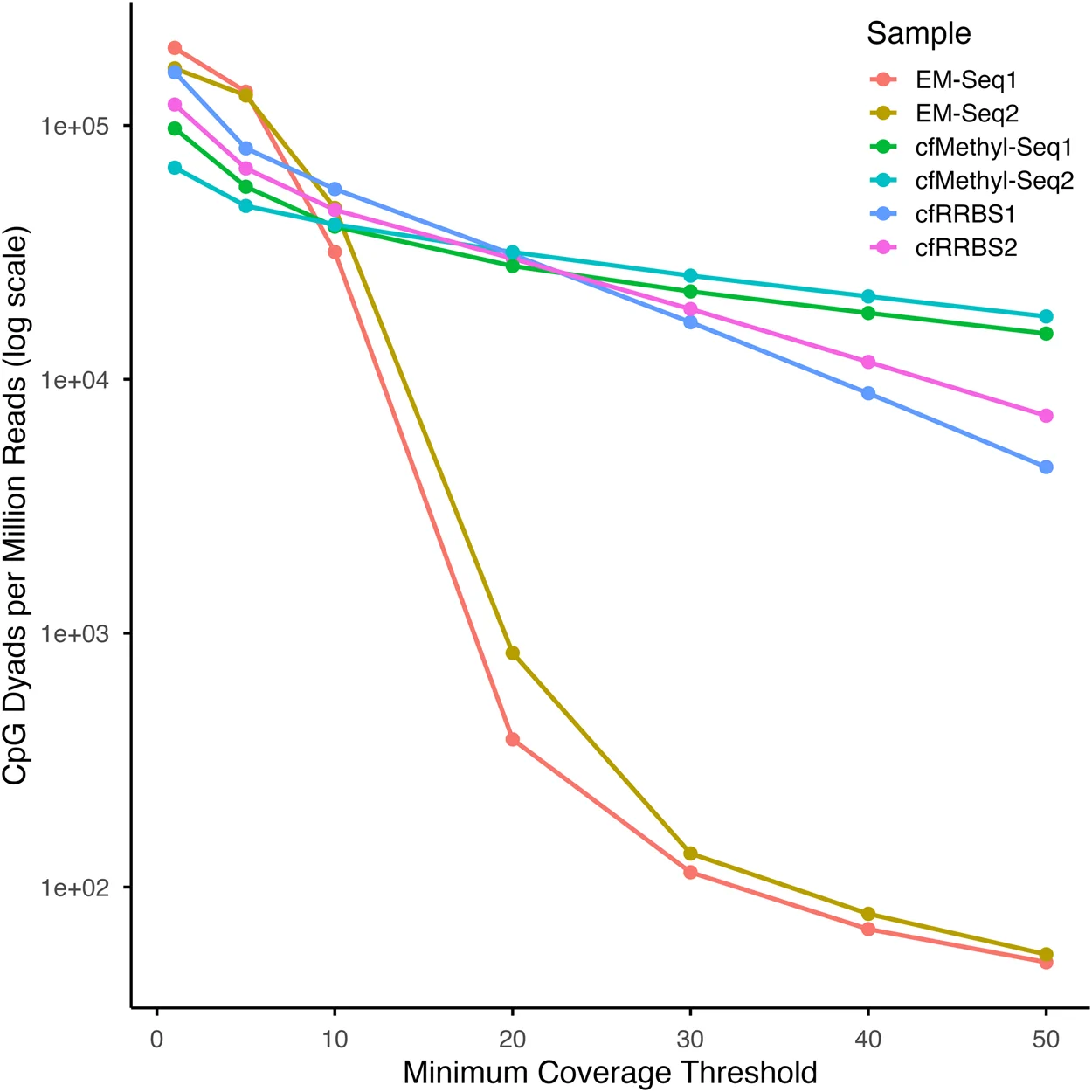
Normalised CpG retention line plot (log-scaled) comparing methods across coverage thresholds. Normalised CpG dyad yield per million mapped reads across coverage thresholds (1x – 50x) for EM-Seq, cfRRBS, and cfMethyl-Seq replicate libraries. CpG counts were corrected for sequencing depth by dividing raw CpG dyad counts at each threshold by the total number of mapped reads per sample (counts-per-million normalisation). The solid lines represent the replicate-averaged CpG retention trend, while the points show the individual replicate measurements. The Y-axis is plotted on a log scale to visualise relative retention dynamics, and the coverage thresholds (X-axis) represent the minimum CpG sequencing depth per site.

To statistically compare the methods, we have applied Welch’s t-test at each coverage level and reported effect sizes (Supplementary Table S1c). At low coverage thresholds (1x – 5x), cfMEthyl-Seq retained significantly fewer CpGs per million reads than EM-Seq (Cohen’s d > 4), while cfRRBS showed comparable performance to EM-Seq. However, at ≥20x coverage thresholds, cfRRBS and cfMethyl-Seq retained substantially more high-confidence CpGs than Em-Seq (d > 10), demonstrating a reversal in efficiency at deeper coverage. Differences between cfRRBS and cfMethyl-Seq were generally small across all thresholds (d < 3).

### Assessment of technical reproducibility

To evaluate technical reproducibility, we focused on high-confidence CpG sites covered at ≥10x coverage threshold across replicates. Within each method, we compared methylation levels between replicate libraries and found varying degrees of agreement. EM-Seq showed modest reproducibility (r = 0.66, 95% CI: 0.662-0.664), with 1.6 million CpGs in common between replicates. In contrast, both bisulfite-based methods demonstrated markedly higher reproducibility. cfRRBS and cfMethyl-Seq demonstrated excellent reproducibility, each achieving a correlation r = 0.97 (95% CI: 0.970 – 0.972), based on 1.1 million and 2.8 million shared CpGs, respectively (Figures 3A–C; Supplementary Table S2). Additionally, the cfRRBS and cfMethyl-Seq libraries exhibited the expected bimodal distributions, whereas EM-Seq is mostly methylated.

**FIGURE 3:**
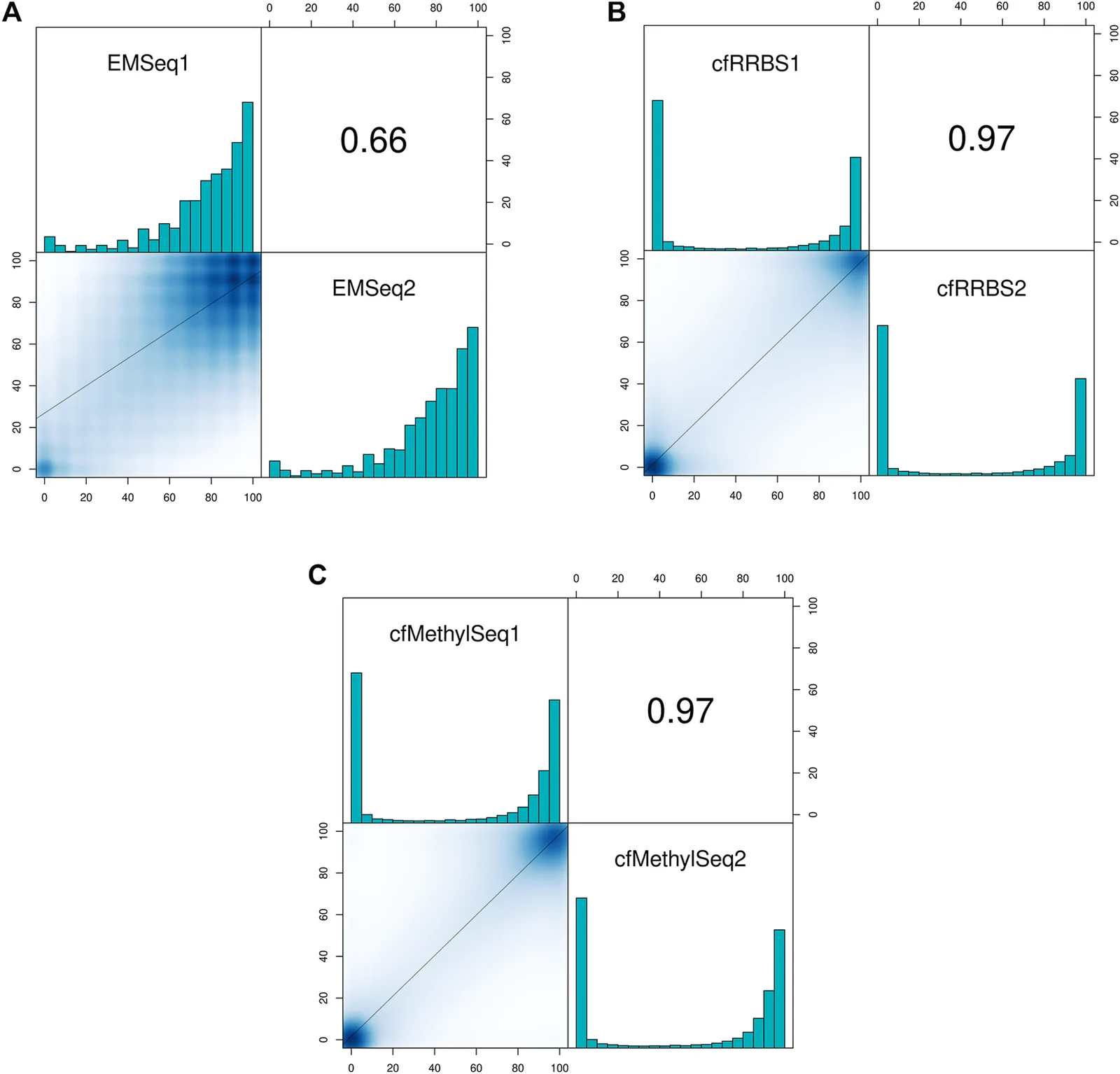
Pearson’s correlation analysis showing the within-platform reproducibility of the **(A)** EM-Seq, **(B)** cfRRBS, **(C)** cfMethyl-Seq replicates. The bars show the methylation distribution across CpG sites for each sample. The scatter plot compares CpG methylation levels across replicates at CpG sites shared at a 10x coverage threshold. Each point represents a single CpG dyad. The solid diagonal line denotes the linear regression fit, indicating the overall concordance pattern between replicates. The dispersion of points around the regression line reflects technical variability in methylation measurement within each method.

To investigate these observed differences further, we compared correlation strength using the Fisher z-transformed effect size, q (Supplementary Table S3). The reproducibility difference between EM-Seq and cfRRBS (q = 1.29) and between EM-Seq and cfMethyl-Seq (q = 1.33) was very large, indicating substantially lower consistency in EM-Seq replicate measurements. In contrast, the difference between cfRRBS and cfMethyl-Seq was negligible (q = 0.03), demonstrating statistically equivalent reproducibility between the two bisulfite-based approaches.

To further assess reproducibility, we applied Bland-Altman analysis to examine the degree of agreement between paired measurements. All methods demonstrated minimal mean bias; however, the extent of variability differed across methods. cfMethyl-Seq exhibited the narrowest limits of agreement (upper limit: 20.6%, lower limit: 20.5%), reflecting highly consistent methylation calls between technical replicates. cfRRBS followed closely with upper and lower limits of agreement at 21.9% and −21.9%, respectively. EM-Seq, on the other hand, showed a broader spread, with upper and lower limits of agreement at 35.6% and −35.6%, respectively, indicating increased technical variability and noise between its replicates (Supplementary Figures S2–S4).

Cross-platform comparisons revealed greater concordance between the bisulfite-based methods. The strongest correlation was observed between cfRRBS and cfMethyl-Seq (r = 0.93). Comparisons involving EM-Seq showed comparatively lower correlations, with r = 0.85 for EM-Seq versus cfRRBS and r = 0.88 for EM-Seq versus cfMethyl-Seq. These comparisons were based on 44,134 common CpG sites across all three methods (Supplementary Figure S5). Comprehensive pairwise assessments are presented in Supplementary Figures S6–S8.

### Genomic region distribution and comparison of methods

Next, we examined the (epi)genomic distribution of the CpGs detected by each method at 10x coverage threshold across replicates (Figure 4). CpGs located within intronic regions were most frequently detected across all methods, but their representation was notably higher in EM-Seq (>60%), compared to approximately 50% for both cfRRBS and cfMethyl-Seq. Exonic regions, by contrast, were more prominent in the reduced representation methods; 20% of cfRRBS-detected CpGs fell within exons (18% for cfMethyl-Seq), double the proportion observed with EM-Seq (8%). Promoter regions also showed enhanced representation with cfRRBS (15%) and cfMethyl-Seq (13%), substantially higher than the 4% captured by EM-Seq.

**FIGURE 4:**
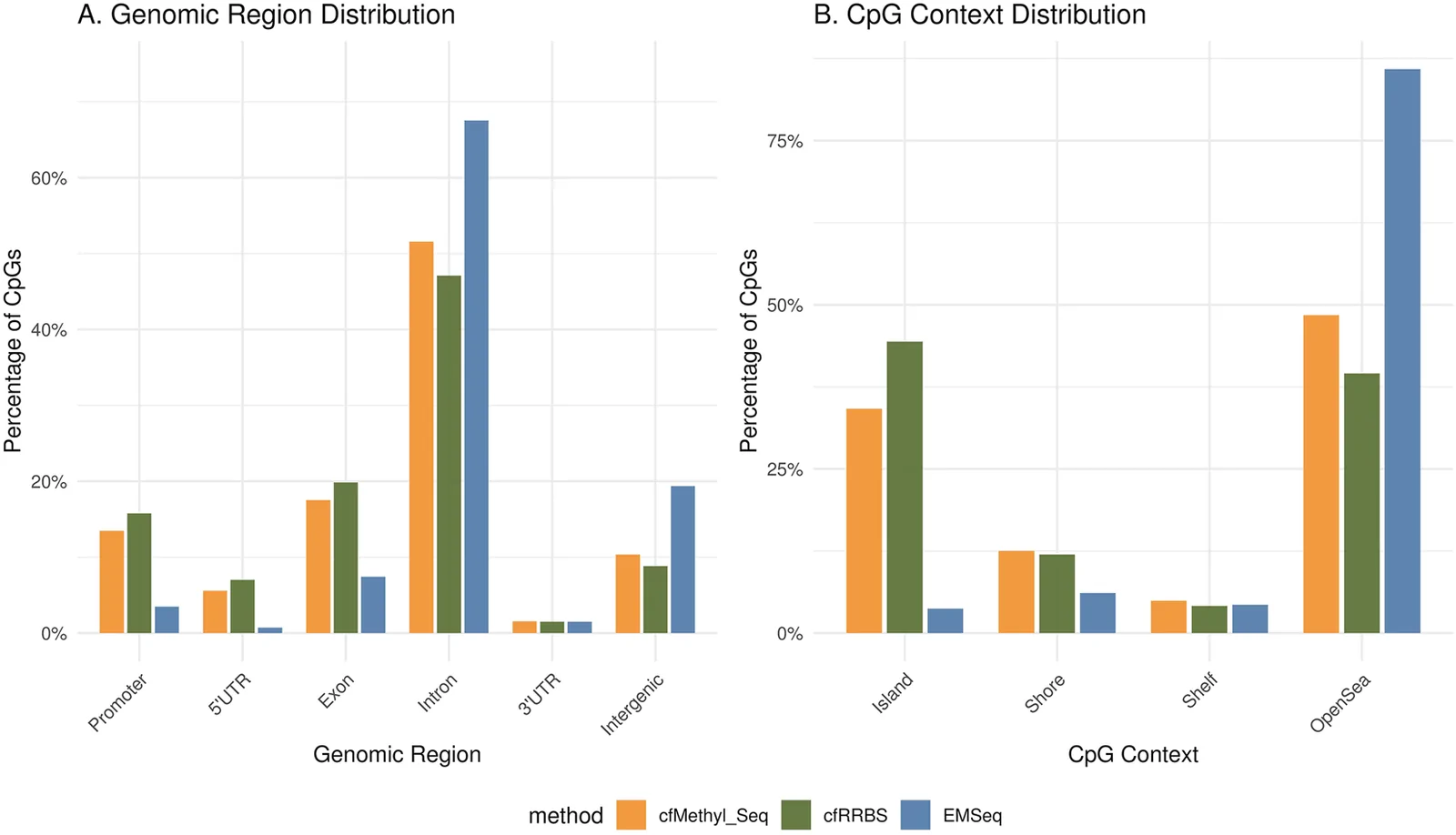
CpG Distribution at key genomic features. **(A)** Distribution of CpG sites across annotated genomic regions (Promoter, UTRs, Exons, Introns, shores, shelves, and intergenic regions) for each DNA methylation profiling method at 10x minimum coverage thresholds. **(B)** CpG context composition showing the proportion of CpGs located in CpG islands, shores, shelves, and open sea regions. Percentages represent the fraction of CpGs detected within each category per method.

From an epigenomic perspective, CpGs in open sea regions dominated the EM-Seq dataset, comprising 82% of CpGs, compared to 38% and 46% for cfRRBS and cfMethyl-Seq, respectively. In contrast, CpG islands were better represented in the reduced-representation methods (45% for cfRRBS and 32% for cfMethyl-Seq), substantially higher than the 5% captured by EM-Seq. These patterns reflect the underlying enrichment strategy for each method, with cfRRBS and cfMethyl-Seq targeting CpG-dense regulatory elements, while EM-Seq provides more uniform whole-genome coverage. Overall, cfRRBS and cfMethyl-Seq showed similar CpG distribution patterns, showing a pronounced bias towards gene regulatory elements.

To determine whether these differences extended beyond discrete promoters to broader regulatory architecture, we investigated the methylation levels around transcription start sites (TSS) and CpG islands (Figure 5). All three methods displayed the expected hypomethylated valleys at regulatory centres, indicating the preservation of promoter-associated demethylation (Vaisvila et al., 2024; Sun et al., 2013). However, EM-Seq maintained consistently higher methylation levels across the full ±1 kb region surrounding both TSS and CpG islands, while cfMethyl-Seq showed intermediate methylation levels more closely aligned with cfRRBS.

**FIGURE 5:**
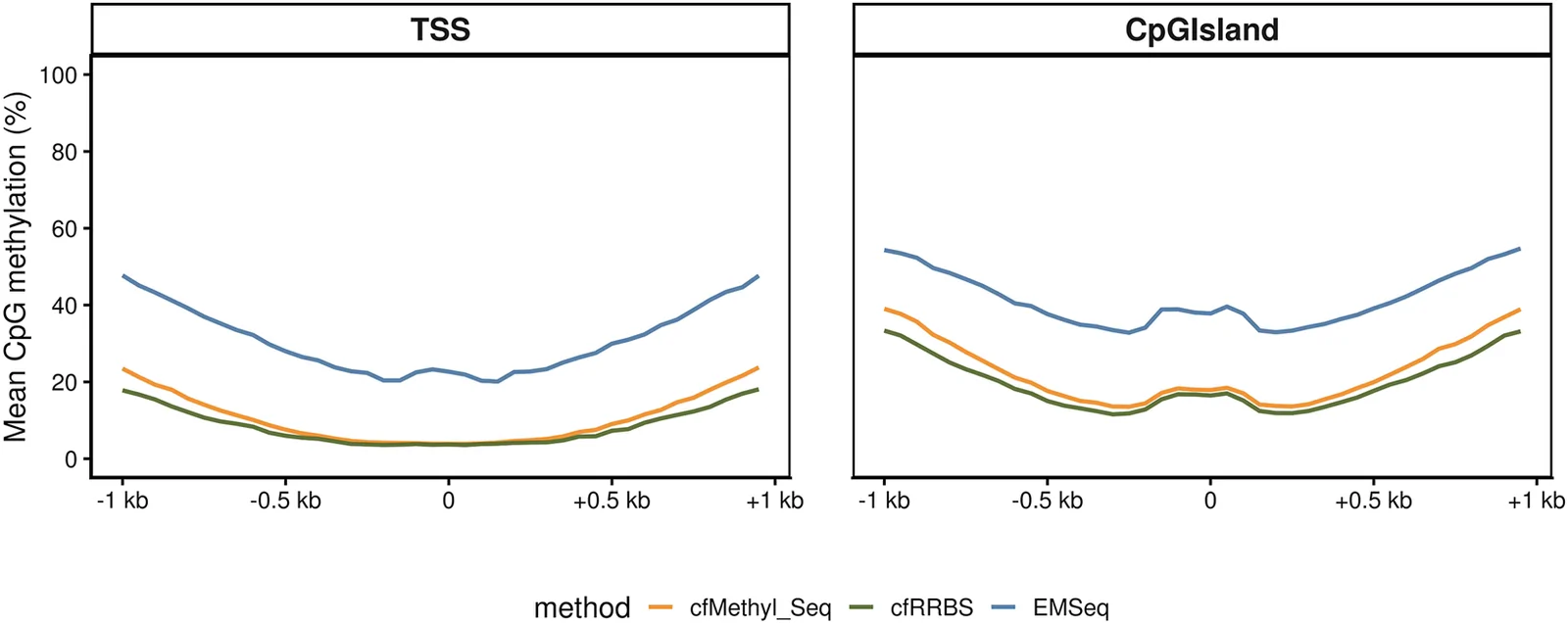
Average CpG methylation profiles around transcription start sites (TSS) and CpG islands across methods. Line plots show mean CpG methylation levels spanning ±1 kb around annotated transcription start sites (left) and CpG islands (right). Curves represent the averaged methylation signal for each method at ≥10x coverage thresholds (EM-Seq, cfRRBS, cfMethyl-Seq), illustrating how cfDNA methylation varies across these genomic features.

To further assess methylation levels at key functional genes, we compared promoter methylation at a panel of ten housekeeping genes. Across the ten genes evaluated, notable differences were observed in the reported methods (Supplementary Tables S4,S5). Mean promoter methylation varied significantly across the three methods (Friedman test p < 0.01; Figure 6A). EM-Seq consistently reported higher methylation values for these genes, frequently exceeding 50%, whereas cfRRBS and cfMethyl-Seq reported values mostly below 30%. Pairwise post-hoc comparisons confirmed that EM-Seq differed significantly from cfMethyl-Seq and cfRRBS (Supplementary Table S6). The heatmap visualisation (Figure 6B) further highlighted the increase in promoter methylation in EM-Seq across nearly all housekeeping genes. Interestingly, EM-Seq exhibited complete promoter coverage across all ten housekeeping genes, whereas cfRRBS and cfMethyl-Seq lacked coverage for the B2M gene (Supplementary Table S4).

**FIGURE 6:**
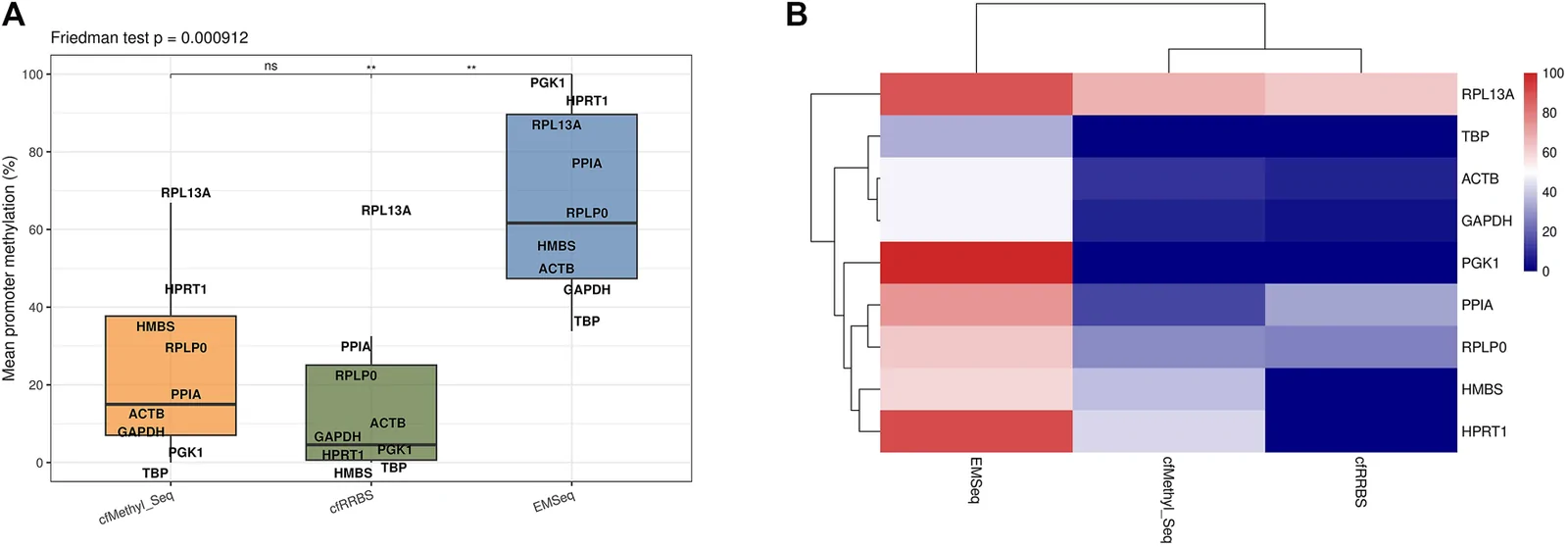
Promoter Methylation at Housekeeping Genes. **(A)** Box-and-label plot showing mean methylation levels for ten constitutively expressed housekeeping genes (GAPDH, ACTB, RPLP0, HPRT1, B2M, TBP, PGK1, HMBS, RPL13A, PPIA), across all methods. Each gene is labelled within its respective method box to show gene-specific variation across methods. A paired Friedman test revealed a significant overall difference across methods (p < 0.01). Dunn’s post-hoc comparisons indicated EM-Seq reported significantly higher promoter methylation than cfRRBS and cfMethyl-Seq, while cfMethyl-Seq and cfRRBS did not differ significantly. **(B)** Heatmap showing promoter methylation (%) for the subset of housekeeping genes with methylation values measured across all three methods. Rows represent genes, and columns represent the library preparation methods. Only the gene promoters present across all methods are shown in the heatmap (See Supplementary Table S4). The values are scaled by raw percentage of methylation (0–100%). Hierarchical clustering highlights consistent elevation of promoter methylation values in EM-Seq, with cfRRBS and cfMethyl-Seq clustering more closely together.

## Discussion

The performance characteristics of enzymatic and bisulfite-based methods for measuring methylation in genomic DNA are well established (Simons et al., 2025; Feng et al., 2020; Morrison et al., 2021); however, a critical gap persists in understanding their performance when applied to cfDNA. Key factors such as DNA integrity, input amount limitations, and the characteristic fragmentation of cfDNA uniquely constrain library preparation efficiency and sequencing performance (Ezegbogu et al., 2024). Our study, therefore, compares the technical performance of enzymatic and bisulfite-based approaches for cfDNA methylation profiling, evaluating key technical performance indicators, including mapping and cytosine conversion efficiencies, proportion of usable data, CpG coverage and genomic distribution, analytical accuracy, reproducibility, and overall cost.

Incomplete cytosine conversion is the primary trigger of biases in methylation studies, with PCR amplification further distorting true methylation signals. PCR-free (or low-cycle-number) protocols are typically preferred for DNA methylation analysis (Olova et al., 2018); however, their utility for cfDNA remains limited due to the inherent challenges associated with low-input material. While the cytosine conversion efficiency in this study was marginally higher for the cfRRBS than for EM-Seq libraries, the former incurs substantial DNA degradation due to bisulfite chemical conversion (Hong and Shin, 2021; Kresse et al., 2025). This limitation is circumvented by EM-Seq, which preserves DNA integrity without overly compromising conversion completeness (Kresse et al., 2025). Notably, our protocol utilised NaOH for DNA denaturation rather than the recommended formamide solution. However, aside from being a less hazardous alternative, there is currently no empirical evidence that formamide improves DNA denaturation (Zon et al., 2009) and, by extension, the efficiency of APOBEC3A activity.

The relatively lower conversion efficiency of the EM-Seq libraries observed in this work agrees with previous studies (Guo et al., 2023), demonstrating that EM-Seq is prone to incomplete conversion in specific genomic contexts, possibly due to the substrate specificity of APOBEC3A deamination (Silvas et al., 2018). Furthermore, compared to standard EM-Seq, the single-enzyme protocol for enzymatic conversion (SEM-Seq) had an even lower conversion efficiency of 96%, particularly in the CH context (Vaisvila et al., 2024). While this limitation may be addressed by bioinformatically removing reads with incomplete conversion (Guo et al., 2023), this approach reduces the proportion of usable data available for downstream analysis.

The mapping efficiency, representing the fraction of reads that align uniquely to the reference genome, is a key determinant of usable data quantity since most alignment software (e.g., Bismark) excludes unmapped and ambiguously-aligned reads (Krueger and Andrews, 2011). In our study, EM-Seq achieved markedly higher mapping efficiency than cfRRBS and cfMethyl-Seq, indicating that EM-Seq provides more informative sequencing reads than the competing methods. The higher mapping efficiency in the EM-Seq libraries is likely driven by two factors: (i) reduced DNA degradation during enzymatic conversion compared to bisulfite treatment (Nuttall et al., 2025; Simons et al., 2025; Kresse et al., 2025), and (ii) the presence of residual unconverted fragments. In bisulfite-based methods, conversion-induced fragmentation generates short reads that map ambiguously to multiple genomic loci and are discarded (Treangen and Salzberg, 2011). In contrast, incomplete enzymatic conversion preserves DNA in its native state, enabling more straightforward alignments without C-to-T transition adjustments. However, such artificial boosts to mapping efficiency do not necessarily reflect improved data quality, as unconverted DNA can introduce false-positive methylation signals if not adequately controlled.

Another metric for assessing usable data in human samples is the number of identified CpGs, as dynamic DNA methylation in humans predominantly occurs at CpG-rich sites (Jones, 2012). While EM-Seq retains more CpGs at lower coverage thresholds than cfRRBS, it exhibits greater coverage-dependent CpG attrition. This is expected, given that EM-Seq targets the whole genome, requiring substantially deeper sequencing, whereas cfRRBS and cfMethyl-Seq focus exclusively on the reduced representation genome^26^. Overall, EM-Seq yielded more usable data, based on its superior mapping efficiency; however, this advantage diminished as the coverage threshold increased, despite costing approximately three times more per sample than cfRRBS. The CpG attrition observed for EM-Seq is due to the distribution of sequencing reads across the genome, with most of them consisting of open sea CpGs. Since these CpG sites are numerous and sparsely distributed, individual sites accumulate depth more slowly, leading to pronounced CpG attrition when stricter thresholds (e.g., ≥10x) are applied. By contrast, cfRRBS and cfMethyl-Seq, which focus on CpG-dense regions, achieve high coverage even with fewer overall reads.

A key consideration in translational clinical research is implementation cost (García-Giménez et al., 2017). Currently, EM-Seq is validated as a whole-genome approach (as opposed to reduced representation), requiring high-depth sequencing to obtain meaningful data. Specific to human samples (excluding embryonic stem cells, brain tissue, and neurons (Lister et al., 2009; Lister et al., 2013)), a substantial proportion of whole-genome methylation data holds limited biological relevance as most dynamic methylation changes occur in CpG-rich regions, while non-CpG contexts (CHG and CHH) exhibit fewer functional variations. The current work shows that EM-Seq provides broader genomic coverage, whereas cfRRBS and cfMethyl-Seq offer focused enrichment of functionally significant regions.

Although EM-Seq could theoretically be adapted for reduced representation, existing strategies have limitations. One approach uses restriction enzymes to deplete AT-rich regions following APOBEC3A conversion (Yang et al., 2024). A similar bisulfite-based approach to generating such CG-rich (or AT-poor) libraries using heat denaturation indicates that this technique may not always measure the same sites in different samples, thus raising questions about its reproducibility (Cheruba et al., 2022). Another option is hybridisation-based capture targeting predetermined CpG sites after enzymatic conversion (Guo et al., 2023). However, this requires *a priori* knowledge of the CpGs of interest, thus making it unsuitable for discovery-phase studies. Moreover, comparative benchmarking of these enzymatic reduced-representation methods against traditional bisulfite sequencing remains lacking.

The correlation analysis offers a clear overview of the relative performance of the library preparation methods. Based on the number of CpG dyads obtained, the bisulfite-based methods demonstrated higher reproducibility (r = 0.97) than EM-Seq (r = 0.66). These results were further supported by Bland-Altman analysis. Both cfRRBS and cfMethyl-Seq showed narrow limits of agreement (±21.88% and ±20.62%, respectively) and minimal bias, indicating consistent methylation measurement across replicates. EM-Seq, on the other hand, exhibited broader limits of agreement (±35.62%) despite a small mean bias, indicating greater variability between technical replicates. These findings are in line with previously reported data from the EM-Seq developers, who observed similar correlations for cfDNA (r = 0.69 (Chaithanya et al., 2019) and r = 0.71 at a 3x coverage threshold) (Vaisvila et al., 2024), but substantially higher reproducibility for genomic DNA (NA12878 cell lines) at a 1x coverage threshold (r = 0.82 (Vaisvila et al., 2021)). This discrepancy suggests that EM-Seq yields more consistent results with high-quality genomic DNA, while performance is more variable with fragmented cfDNA.

Although we did not directly assess fragment length distribution here, the observed coverage profiles suggest that the combination of cfDNA fragmentation and whole-genome sampling influences depth uniformity in EM-Seq libraries. Previous benchmarking studies have shown that EM-Seq has a stable and consistent genomic coverage, but may yield broader variance across CpG-dense elements (Vaisvila et al., 2021; Vaisvila et al., 2024; Foox et al., 2021). Future work integrating fragmentomic analysis will be essential to determine how fragmentation patterns and genomic context jointly influence CpG recovery and reproducibility, particularly in the context of cfDNA.

Our comparative analysis of CpG genomic distribution highlights key methodological biases inherent to each library preparation approach. The substantial detection of intronic and open-sea CpGs by EM-Seq aligns with expectations for whole-genome coverage methods, which provide a more uniform but less targeted representation of regulatory elements. In contrast, cfRRBS and cfMethyl-Seq demonstrated strong enrichment for CpGs within promoters, 5′ UTRs, exons, and CpG islands, consistent with reduced-representation design that preferentially samples CpG-dense and functionally relevant genomic regions (Gu et al., 2011). Such enrichment may enhance the detection of biologically significant methylation changes, particularly in promoter and CpG island contexts, which are often implicated in gene regulation and disease-associated epigenetic alterations.

The observed elevation in promoter methylation, as reported by EM-Seq, has important implications for biological interpretation. Promoters of housekeeping genes are typically hypomethylated in healthy tissues and provide a key mechanism for transcription (Chatterjee et al., 2018). Even modest changes in methylation at these loci can influence transcriptional output and are often associated with altered cell identity or pathological epigenetic remodelling (Bird, 2002). In our analysis, EM-Seq consistently produced higher methylation estimates at these CpG-dense housekeeping promoters compared to cfRRBS and cfMethyl-Seq. This systematic upward shift aligns with benchmarking studies that have reported elevated methylation calls in CpG-rich regions in EM-Seq compared to bisulfite-based approaches (Guanzon et al., 2024; Han et al., 2022). However, other comparative studies have not detected differences in promoter methylation between EM-Seq and bisulfite-based methods (de Abreu et al., 2025), indicating that the magnitude and direction of method biases may depend on experimental conditions (Bird, 2002). Differences in library construction, notably DNA shearing and the timing of adapter ligation (i.e., before or after bisulfite conversion), may explain the differences in methylation states at CpG-dense regions across methods; these variables have yet to be systematically investigated (Morrison et al., 2021).

Differences in genomic footprint also influenced locus detectability. EM-Seq provided complete promoter coverage across all ten housekeeping genes, whereas cfRRBS and cMethyl-Seq did not detect CpGs within the B2M promoter. This reflects the distinct biochemical and enrichment strategies underlying these methods. EM-Seq performs genome-wide enzymatic conversion without locus-specific enrichment, while cfRRBS and cfMethyl-Seq interrogate CpG-rich fragments via restriction digestion. In the context of biomarker discovery, this distinction highlights an important trade-off. A broader footprint increases the likelihood of capturing candidate regulatory loci, but as shown in our results, EM-Seq also exhibited systematically elevated methylation levels in promoter regions expected to remain unmethylated (or hypomethylated) (Tung et al., 2024; Eisenberg and Levanon, 2013). Conversely, the two bisulfite-based methods demonstrated more accurate low-methylation baselines at these loci, but at the cost of reduced locus availability when promoters fall outside their enrichment targets. Thus, loci detectability and quantitative accuracy do not necessarily coincide, and method selection for diagnostic assay development must balance these parameters rather than optimise one at the expense of the other.

However, it is important to contextualise these findings. The majority of comparative evaluations of EM-Seq versus bisulfite conversion have been conducted in high-molecular-weight genomic DNA derived from cultured cells or bulk tissues. In contrast, cell-free DNA exhibits distinct fragmentomic patterns, nucleosomal positioning biases, and differential sequence protection (Snyder et al., 2016; Swarup et al., 2025); all of which can interfere with library preparation chemistry. Consequently, the direction of method-dependent methylation shifts observed in cfDNA may not fully mirror those reported in genomic-DNA-based benchmarking studies. Further systematic comparisons utilising standardised cfDNA reference materials and orthogonal controls will be required to determine whether the promoter hypermethylation shift described here reflects true biological signal, differential fragment recovery, or method-specific detection biases in the cell-free DNA context.

The comparable distribution patterns observed between cfRRBS and cfMethyl-Seq indicate that differences in their CpG enrichment chemistries do not substantially alter the genomic contexts captured, reinforcing their suitability for methylation studies in the cfDNA context. Based on our observations in this work, the unique features of EM-Seq, cfRRBS, and cfMEthyl-Seq are summarised in Table 2.

**TABLE 2:** Unique Features/Considerations of EM-Seq, cfRRBS, and cfMethyl-Seq.

Parameter	EM-seq	cfRRBS	cfMethyl-seq
^a^DNA loss	Multiple tubes and bead clean-up protocols	Single-tube experiment and one bead clean-up protocol	Multiple tubes and bead clean-up step protocols
PCR	Low number of PCR cycles (D et al., 2021; Ezegbogu et al., 2024; Allen Chan et al., 2013; Baylin et al., 2001; Esteller et al., 2024); minimises the risk of PCR bias	High number of PCR cycles (Beck et al., 2021; Deaton and Bird, 2011; Meissner et al., 2005); increases the potential for PCR bias	High number of PCR cycles (Beck et al., 2021; Deaton and Bird, 2011; Meissner et al., 2005); mitigated by the inclusion of UMIs^b^
Control materials	Includes unmethylated lambda and CpG-methylated pUC19 DNA controls for assessing conversion efficiency	Includes unmethylated lambda DNA control	No control materials included
Technical Support	Company-developed method. Technical support is guaranteed	Technical support may be available (Koker et al., 2019)	Technical support may be available (Stackpole et al., 2022)
Minimum Input DNA	3 ng (10 pg for genomic DNA) (Vaisvila et al., 2024)	0.7 ng (Schoofs et al., 2024)	5 ng (Stackpole et al., 2022)
^c^Sequencing depth	Requires deep sequencing to obtain meaningful data	Low sequencing depth sufficiently covers the RRBS genome	Low sequencing depth sufficiently covers the RRBS genome
Application	Amenable to a wide array of sample types, e.g., mammalian and plant DNA	Applicable to mammalian samples and could potentially be adopted for other species (e.g., Zebrafish (Chatterjee et al., 2016))	Applicable to mammalian samples and could potentially be adopted for other species

^a^ Bead purification increases opportunities for DNA loss; however, cfDNA recovery efficiency depends substantially on the user’s skill level.

^b^ UMIs: Unique Molecular Identifiers.

^c^ Approximately 90 Gigabytes of data provides a 70x coverage of the RRBS genome (∼3.05 GB), while 5 Gigabytes of sequencing data provides a 70x coverage of the RRBS, genome (70 Mb).

While this paper was in preparation, a related study by Kresse et al. (2025) was published, comparing enzymatic and bisulfite conversion methods. Despite adopting different technical approaches, both studies report comparable conversion efficiencies (Kresse et al. used targeted ddPCR to evaluate the bisulfite conversion efficiency, whereas we employed a spike-in lambda control). While their findings highlight the clinical advantages of bisulfite-based conversion in cancer diagnosis, our study extends this observation by demonstrating that cfRRBS preferentially measures functionally relevant genomic regions. In contrast, EM-Seq, while detecting more CpGs, primarily captures sites in open sea regions with limited functional significance, especially in biomarker discovery. We also note that EM-Seq demonstrates greater genomic coverage than the bisulfite-based methods evaluated in this work.

This study provides a controlled technical comparison of enzymatic and bisulfite-based methods for cfDNA methylation profiling; however, several limitations should be noted. First, only two technical replicates were processed per method. This design was intentional as it enabled a focused evaluation of technical performance and reproducibility while minimising the confounding effects of inter-individual biological variation. Nevertheless, this limited sample size constrains the biological generalizability of our findings. Accordingly, the results presented here should be interpreted as a technical performance benchmark rather than a definitive characterisation of cfDNA biology. Future studies involving larger, well-powered cohorts that capture the biological heterogeneity encountered in clinical settings will be essential to confirm the clinical utility and diagnostic potential of these approaches.

Second, although sequencing depth differed across methods, an equivalent depth was not required to evaluate the cfRRBS libraries, as their genomic footprint is inherently restricted. Nonetheless, where depth directly impacts interpretability, such as CpG retention, we applied normalisation to ensure method-to-method comparability. Third, the study contrasts whole-genome sampling (EM-Seq) with reduced-representation enrichment strategies (cfRRBS and cfMethyl-Seq). These methodological philosophies are fundamentally different, and the trade-offs we document should be interpreted within this context rather than as deficits of any individual platform.

Finally, the cfDNA samples were obtained from self-reported healthy volunteers without independent verification of clinical status. While this does not affect the technical comparisons presented here, future validation studies should involve rigorously phenotyped cohorts.

Importantly, the insights gained from this work suggest a promising avenue for method refinement. For example, combining reduced-representation enrichment strategies (e.g., cfRRBS or cfMethyl-Seq) with enzymatic cytosine conversion (e.g., NEBNext® Enzymatic Methyl-Seq Conversion Module (New England Biolabs)). Such hybrid approaches may integrate the coverage efficiency of enrichment with the reduced damage associated with enzymatic conversion, potentially improving both sensitivity and reproducibility in cfDNA methylome assays used for biomarker discovery.

## Conclusion

In summary, this study systematically benchmarks enzymatic and bisulfite conversion protocols for cfDNA methylation analysis. While our results are specific to the kits and protocols used here, they highlight distinct advantages of each method: EM-Seq achieves superior mapping efficiency, thus preserving more sequencing reads for downstream analysis, whereas cfRRBS and cfMethyl-Seq offer higher reproducibility, higher cytosine conversion efficiency, and reduced coverage-dependent CpG attrition, more so, at significantly lower cost per sample. Moreover, the bisulfite-based approaches preferentially enrich for functionally relevant genomic regions, offering an advantage for biological interpretation. These findings provide a practical framework for method selection in liquid biopsy studies involving cfDNA methylation assays.

## Data Availability

The original contributions presented in the study are publicly available. This data can be found here: https://www.ncbi.nlm.nih.gov/geo/query/acc.cgi?acc=GSE306695.
