# Supplementary material for: Comparison of enzymatic and bisulfite-based methods for sequencing-based cell-free DNA methylation profiling
Source: Front Epigenet Epigenom. 2026 Jan 16;3:1693925. doi: 10.3389/freae.2025.1693925 (PMC13639271; doi:10.3389/freae.2025.1693925)
Supplement: Supplementary file 1 [file DataSheet1.docx]

Supplementary Table 1a

Raw CpG Dyad Count for the Sample Replicates

| **Sample** | **Number of Reads** | **1x** | **5x** | **10x** | **20x** | **30x** | **40x** | **50x** |
| --- | --- | --- | --- | --- | --- | --- | --- | --- |
| **EM-Seq1** | 133,534,259 (PE) | 26,974,281 | 18,122,390 | 4,240,443 | 50,978 | 15,270 | 9,122 | 6,756 |
| **EM-Seq2** | 162,049,688 (PE) | 27,193,494 | 21,268,651 | 7,668,898 | 135,511 | 21,983 | 12,717 | 8,802 |
| **cfRRBS1** | 23,391,363 (SE) | 3,787,223 | 1,901,063 | 1,313,954 | 725,692 | 392,564 | 206,029 | 105,593 |
| **cfRRBS2** | 30,906,423 (SE) | 3,735,889 | 2,092,425 | 1,437,594 | 923,751 | 584,879 | 361,825 | 222,162 |
| **cfMethyl-Seq1** | 80,246,399 (PE) | 7,807,315 | 4,602,331 | 3,204,919 | 2,242,205 | 1,780,210 | 1,464,250 | 1,215,044 |
| **cfMethyl-Seq2** | 83,643,744 (PE) | 5,703,106 | 4,030,977 | 3,407,996 | 2,649,320 | 2,141,637 | 1,774,171 | 1,478,909 |

Supplementary Figure 1: CpG dyad count comparing methods across coverage thresholds.

Supplementary Table 1b

Normalised CpG Dyad Count per million sequencing reads for the sample replicates

| **Sample** | **1x_perM** | **5x_perM** | **10x_perM** | **20x_perM** | **30x_perM** | **40x_perM** | **50x_perM** |
| --- | --- | --- | --- | --- | --- | --- | --- |
| **EM-Seq1** | 202,003 | 135,713 | 31,755 | 382 | 114 | 68 | 51 |
| **EM-Seq2** | 167,810 | 131,248 | 47,324 | 836 | 136 | 78 | 54 |
| **cfRRBS1** | 161,907 | 81,272 | 56,173 | 31,024 | 16,782 | 8,808 | 4,514 |
| **cfRRBS2** | 120,877 | 67,702 | 46,514 | 29,889 | 18,924 | 11,707 | 7,188 |
| **cfMethyl-Seq1** | 97,292 | 57,352 | 39,938 | 27,942 | 22,184 | 18,247 | 15,141 |
| **cfMethyl-Seq2** | 68,183 | 48,192 | 40,744 | 31,674 | 25,604 | 21,211 | 17,681 |

Supplementary Table 1c

CpG retention efficiency normalised per million sequencing reads across cfDNA methylation methods

| Coverage Threshold | comparison | t_stat | p_value | cohens_d |
| --- | --- | --- | --- | --- |
| 1x | cfRRBS vs EM-Seq | -1.62944 | 0.248747 | -1.62944 |
|  | cfMethyl-Seq vs EM-Seq | -4.55042 | 0.047216 | -4.55042 |
|  | cfRRBS vs cfMethyl-Seq | -2.3319 | 0.158561 | 2.331901 |
| 5x | cfRRBS vs EM-Seq | -8.25895 | 0.051487 | -8.25895 |
|  | cfMethyl-Seq vs EM-Seq | -15.8394 | 0.01339 | -15.8394 |
|  | cfRRBS vs cfMethyl-Seq | -2.65258 | 0.134439 | 2.65258 |
| 10x | cfRRBS vs EM-Seq | 1.288508 | 0.347435 | 1.288508 |
|  | cfMethyl-Seq vs EM-Seq | 0.102813 | 0.934708 | 0.102813 |
|  | cfRRBS vs cfMethyl-Seq | -2.27042 | 0.261387 | 2.270421 |
| 20x | cfRRBS vs EM-Seq | 48.81495 | 0.004235 | 48.81495 |
|  | cfMethyl-Seq vs EM-Seq | 15.53154 | 0.03796 | 15.53154 |
|  | cfRRBS vs cfMethyl-Seq | -0.33252 | 0.788723 | 0.332516 |
| 30x | cfRRBS vs EM-Seq | 16.55411 | 0.03839 | 16.55411 |
|  | cfMethyl-Seq vs EM-Seq | 13.90001 | 0.045713 | 13.90001 |
|  | cfRRBS vs cfMethyl-Seq | 2.994093 | 0.117691 | -2.99409 |
| 40x | cfRRBS vs EM-Seq | 7.025414 | 0.090008 | 7.025414 |
|  | cfMethyl-Seq vs EM-Seq | 13.26228 | 0.047909 | 13.26228 |
|  | cfRRBS vs cfMethyl-Seq | 4.568695 | 0.044761 | -4.56869 |
| 50x | cfRRBS vs EM-Seq | 4.337084 | 0.144263 | 4.337084 |
|  | cfMethyl-Seq vs EM-Seq | 12.88277 | 0.049317 | 12.88277 |
|  | cfRRBS vs cfMethyl-Seq | 5.726948 | 0.029339 | -5.72695 |

CpG dyad counts at coverage thresholds (1x – 50x) were normalised to counts per million reads to control for sequencing depth. Welch’s test (two-tailed) was performed to compare CpG retention efficiency between methods at each threshold. Due to the small number of biological replicates (n = 2), the effect size (Cohen’s d) was reported to quantify the magnitude of differences.

Supplementary Table 2

Technical reproducibility of cfDNA methylation profiling methods at 10x coverage thresholds

| Method | r (Pearson) | N_pairs | 95% CI (lower) | 95% CI (upper) |
| --- | --- | --- | --- | --- |
| EM-Seq | 0.66 | 1600000 | 0.659 | 0.661 |
| cfRRBS | 0.97 | 1100000 | 0.970 | 0.970 |
| cfMethyl-Seq | 0.97 | 2800000 | 0.970 | 0.970 |

Pearson correlation coefficients (r) were calculated between technical replicate libraries for each platform at CpG sites with >10x coverage. N represents the number of CpG sites shared between replicate pairs used in the correlation analysis. 95% confidence intervals were computed using Fisher’s z-transformation.

Supplementary Table 3: Cross-platform Reproducibility Comparisons at 10x coverage thresholds

| Comparison | Fisher’s z statistic | Two-tailed p-value | Cohen’s q (effect size) |
| --- | --- | --- | --- |
| cfRRBS vs EM-Seq | 1059 | < 2 x 10^-16^ | 1.29 |
| cfMethyl-Seq vs EM-Seq | 1351 | < 2 x 10^-16^ | 1.33 |
| cfRRBS vs cfMethyl-Seq | -29.9 | < 2 x 10^-16^ | 0.03 |

Fisher’s z-tests were used to compare correlations between methods, using the number of shared CpGs to weight the z-transformed correlation differences. Two-tailed p-values are reported using log scale correction due to the large CpG counts. Cohen’s q is presented as an effect size measure of the difference in correlation strength. Effect sizes >0.50 indicate a large difference, whereas effect sizes <0.10 indicate a negligible difference.

Supplementary Figure 2: Bland-Altman plot showing the EM-Seq technical replicates.

Mean difference (bias): -0.01

Upper limit of agreement: 35.63

Lower limit of agreement: -35.64

Supplementary Figure 3: Bland-Altman plot showing the cfRRBS technical replicates.

Mean difference (bias): 0.001

Upper limit of agreement: 21.88

Lower limit of agreement: -21.88

Supplementary Figure 4: Bland-Altman plot showing the cfMethyl-Seq technical replicates.

Mean difference (bias): 0.05

Upper limit of agreement: 20.62

Lower limit of agreement: -20.53

Supplementary Figure 5: Correlation of EM-Seq vs cfRRBS vs cfMethyl-Seq at 10x coverage threshold. The replicates have 44,134 common CpGs.

Supplementary Figure 6: Correlation of EM-Seq vs cfRRBS at 10x coverage threshold. The replicates have 45,664 common CpGs.

Supplementary Figure 7: Correlation of EM-Seq vs cfMethyl-Seq at 10x coverage threshold. The replicates have 125,808 common CpGs.

Supplementary Figure 8: Correlation of cfRRBS vs cfMethyl-Seq at 10x coverage threshold. The replicates have 1,099,844 common CpGs.

Supplementary Table 4: Mean CpG Methylation Across Housekeeping Gene Promoters

| Gene | cfMethyl-Seq (%) | cfRRBS (%) | EM-Seq (%) |
| --- | --- | --- | --- |
| ACTB | 10.8 | 7.56 | 47.4 |
| GADPH | 7.03 | 4.57 | 47.2 |
| HMBS | 37.7 | 0.00 | 58.5 |
| HPRT1 | 42.0 | 0.538 | 90.6 |
| PGK1 | 0.00 | 1.08 | 100.0 |
| PPIA | 15.0 | 32.5 | 74.5 |
| RPL13A | 66.9 | 62.4 | 89.6 |
| RPLP0 | 27.0 | 25.1 | 61.7 |
| TBP | 0.00 | 0.641 | 33.8 |
| B2M | NA | NA | 80.0 |

Average methylation levels were calculated for CpGs located within promoter regions of ten housekeeping genes, defined as -1 kb upstream to +5 kb downstream of the annotated TSS (hg19). Values represent mean beta methylation percentages for each method following replicate merging and coverage depth filtering (≥10x). Genes with missing values (NA) reflect a lack of CpG coverage in the specified promoter region for that method.

Supplementary Table 5: Friedman Test for Housekeeping Promoter Methylation

|  | n | Statistic (X^2^) | df | p-value | Test |
| --- | --- | --- | --- | --- | --- |
| Mean_meth | 9 | 14.0 | 2 | 0.000912 | Friedman test |

This table shows the results of a Friedman paired non-parametric test assessing whether promoter methylation levels at ten housekeeping genes differ across the three cfDNA methylation profiling methods (cfMethyl-Seq, cfRRBS, and EM-Seq). Each housekeeping gene contributes one paired measurement per method. n denotes the number of genes included after filtering for complete data across methods. A significant p-value indicates that at least one method reports systematically different methylation levels across these promoters.

Supplementary Table 6: Dunn Post-hoc Test Comparing Promoter Methylation between Methods.

| Comparison | Method_1 | Method_2 | n | Statistic | p | p.adj | Significance |
| --- | --- | --- | --- | --- | --- | --- | --- |
| Mean_meth | cfMethyl-Seq | cfRRBS | 9 | -0.624 | 0.533 | 0.533 | ns |
| Mean_meth | cfMethyl-Seq | EM-Seq | 9 | 2.76 | 0.00572 | 0.00858 | ** |
| Mean_meth | cfRRBS | EM-Seq | 9 | 3.39 | 0.000706 | 0.00212 | ** |

Post-hoc pairwise Wilcoxon rank comparisons were performed following the Friedman test, with Benjamini-Hochberg correction applied to control FDR. p.adj denotes the adjusted p-value, and p.adj corresponds to significance coded as: ns = not significant; p<0.05 = *; p<0.01 = **; p< 0.001 = ***; p<0.0001 = ****.
